# Response inhibition on the stop signal task improves during cardiac contraction

**DOI:** 10.1038/s41598-018-27513-y

**Published:** 2018-06-14

**Authors:** Charlotte L. Rae, Vanessa E. Botan, Cassandra D. Gould van Praag, Aleksandra M. Herman, Jasmina A. K. Nyyssönen, David R. Watson, Theodora Duka, Sarah N. Garfinkel, Hugo D. Critchley

**Affiliations:** 1 Sackler Centre for Consciousness Science, University of Sussex, Falmer, UK; 2 Department of Neuroscience, Brighton & Sussex Medical School, Falmer, UK; 3 School of Psychology, University of Sussex, Falmer, UK; 4 Sussex Partnership NHS Foundation Trust, Brighton, UK

## Abstract

Motor actions can be facilitated or hindered by psychophysiological states of readiness, to guide rapid adaptive action. Cardiovascular arousal is communicated by cardiac signals conveying the timing and strength of individual heartbeats. Here, we tested how these interoceptive signals facilitate control of motor impulsivity. Participants performed a stop signal task, in which stop cues were delivered at different time points within the cardiac cycle: at systole when the heart contracts (T-wave peak, approximately 300 ms following the R-wave), or at diastole between heartbeats (R-wave peak). Response inhibition was better at systole, indexed by a shorter stop signal reaction time (SSRT), and longer stop signal delay (SSD). Furthermore, parasympathetic control of cardiovascular tone, and subjective sensitivity to interoceptive states, predicted response inhibition efficiency, although these cardiovascular and interoceptive correlations did not survive correction for multiple comparisons. This suggests that response inhibition capacity is influenced by interoceptive physiological cues, such that people are more likely to express impulsive actions during putative states of lower cardiovascular arousal, when frequency and strength of cardiac afferent signalling is reduced.

## Introduction

The ability to stop or prevent an inappropriate response is a fundamental feature of adaptive behaviour. Motor responses and their inhibitory control are affected by states of psychophysiological motor readiness, and correspondingly, the control of these states through central and peripheral (including autonomic) nervous activity. Changes in internal physiological arousal may serve as precognitive ‘markers’ of risk to guide adaptive behaviour^1,2^ including rapid aversion responses that can encompass cancelling an action already in preparation. Natural fluctuations in internal state, notably the feedback from individual heartbeats, impact the sensory processing of salient events^3^, changing thresholds for the rapid detection of fear and threat stimuli^4^. Similarly, these physiological afferent cues may also facilitate more rapid motor reactions to salient events in support of adaptive behaviour.

Cardiovascular afferents are a potent conduit of interoceptive information concerning physiological states relevant to motivational behaviour. With each heartbeat, the ejection of blood into the aorta and carotid arteries activates arterial baroreceptors. Their phasic firing is relayed to brainstem nuclei, conveying the timing and strength of each cardiac contraction, thus encoding heart rate and blood pressure, namely, the state of cardiovascular arousal^3^. From the brainstem, this information ascends to basal ganglia and cortex, in particular insular and cingulate regions sensitive to behavioural salience^5–7^. Therefore, cortical centres have rapid access to physiological information about the state of internal homeostatic integrity, and can trigger mitigating responses through autonomic adjustment or motor action^2,5^.

The cardiac cycle impacts behaviour and cognition: brief sensory events occurring at discrete cardiac phases (i.e. at systole or diastole) may be processed by differently, as seen for pain responses^8^, memory^9,10^, and emotional processing^4^. Furthermore, motor acts in simple reaction time tasks can be influenced by cardiac state^11,12^. During states of cardiovascular arousal, heart rate and blood pressure rise together, ensuring proportionally more time is spent in ventricular systole. This results in stronger and more sustained activation of arterial baroreceptors. Consequently, the effects of this afferent channel on the likelihood of detecting, and responding to, certain salient events is amplified during states of cardiovascular arousal^3^. Furthermore, the effects of systolic baroreceptor firing on sensorimotor behaviour may be predominantly inhibitory, and historically this was assumed to be the only effect^13^. However, the systolic facilitation of fear processing represents one important exception^4^.

These findings raise the question of whether phasic interoceptive signals concerning cardiovascular arousal influence the efficiency with which appropriate action responses are generated or selected. Importantly, this extends to the inhibition of initiated actions. An everyday example is the need to cancel a step to cross the road when a vehicle appears unexpectedly. This form of response inhibition is modelled in the laboratory by the stop signal task, in which pre-potent cues to ‘go’ are occasionally followed by a ‘stop’ cue, instructing the participant to abort the action^14^.

The stop signal task was previously applied to examine the effect of response inhibition on heart rate adjustments to action processes: for example, cardiac deceleration or acceleration is observed during action preparation or action execution respectively^15^. During successful action inhibition, cardiac deceleration is maintained, delaying the ‘acceleratory recovery’^16,17^. However, these findings do not examine how timing of salient action cues at points within the cardiac cycle can affect response inhibition.

Here, we focus on the effect that physiological signals concerning cardiovascular arousal have on action, rather than the effect that action control has on physiology. Specifically, we test whether stop cues delivered at systole, when the heart is contracting, or at diastole, between heartbeats, change response inhibition efficiency, as indexed by the stop signal reaction time (SSRT). We predicted that physiological arousal signals at systole prompt more rapid responses to stop cues, given the prioritized detection of salient events during heightened cardiovascular arousal^3^, and the adaptive function of homeostatic systems to drive avoidant and mitigating behaviours^2,5^.

Furthermore, individual differences in stop signal task performance reflect cardiac physiology^18^ and trait or neurobehavioural ‘endophenotype’^19^. We therefore tested if the impact of interoceptive arousal signals (within the cardiac cycle) relate to baseline autonomic differences in sympathetic-parasympathetic balance, and impulsivity and interoceptive endophenotypes.

## Results

Sixty participants gave written informed consent. Eight participants were excluded for failing to follow instructions not to wait for the stop signal, evidenced by long go reaction times and/or long stop signal delay (SSD) values (more than 2 standard deviations from the group mean). A further six participants were excluded because one of the two staircase trackers to adjust the SSD failed to converge sufficiently to approximately 50% (mean inhibition success more than 2 standard deviations from the group mean). Data are presented from the remaining 46 participants (21 male; age 18–31 years, mean 23 years).

### Cardiac Stop Signal Task

**Figure 1:**
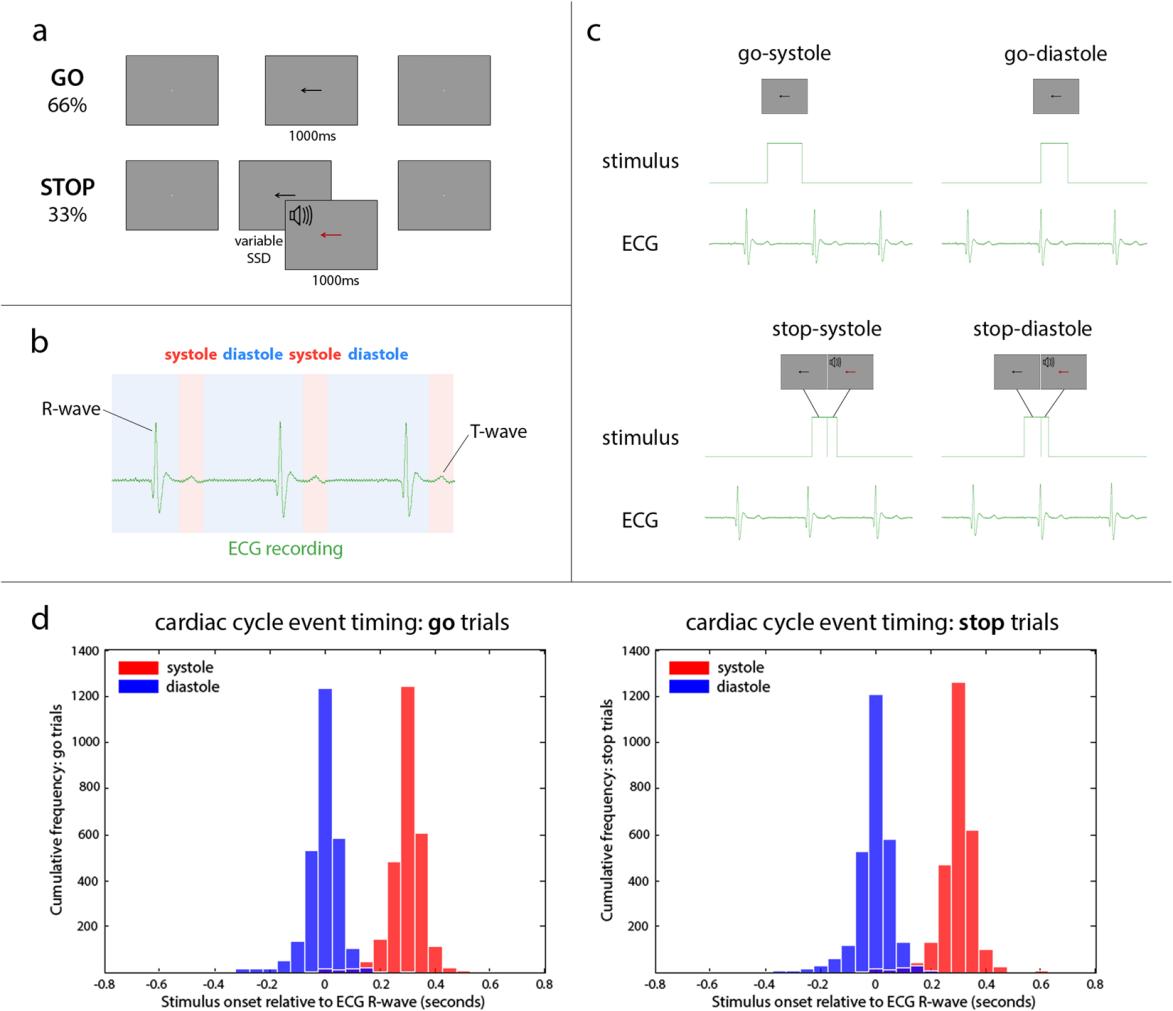
Cardiac stop signal task. (**a**) Stop signal task, (**b**) cardiac cycle in relation to ECG signal: systole (cardiac contraction) occurs around the T-wave, which is typically ~300 ms after the R-wave, (**c**) cardiac timing of stop signal task event onsets, (**d**) precision of trial event timing within the cardiac cycle, relative to the R-wave peak, in 50 ms time bins: >90% of trials were within 200 ms of the intended timing for diastole trials at 10 ms prior to the R-wave, and for systole trials at 290 ms following R-wave. Dark blue indicates overlap of diastole and systole trial timings (minimal for both go and stop trials).

Participants performed a stop signal task (Fig. 1a) in which the onset of the stop cues was timed to either cardiac systole (when the heart is contracting) or cardiac diastole (when the heart is relaxed between beats). To synchronise onset of the stop cues to specific time points within the cardiac cycle (Fig. 1b), we used ECG recording, interfacing cardiac events with the task in Matlab. Using the R-wave peak, the relevant stimulus was delivered to coincide either with cardiac systole (corresponding to the T-wave peak of the ECG), at 290 ms following R-wave peak; or cardiac diastole, at 10 ms prior to the R-wave peak (Fig. 1c).

The precision of trial event timing within the cardiac cycle, relative to the R-wave peak (Fig. 1d) was such that >90% of trials were within 200 ms of the intended timing for both systole (red) and diastole (blue) trials, with minimal overlap (dark blue). The mean timing relative to the R-wave peak for systole trials was 296 ms for go trials (standard deviation = 70 ms), and 296 ms for stop trials (SD = 74 ms). Within stop-systole trials, the mean timing for stop success trials (response withheld) was 296 ms (SD = 75 ms), and for stop fail trials (button pressed) was 296 ms (SD = 72 ms) (Supplementary Figure 1). On diastole trials, the mean timing relative to the R-wave peak was −7 ms for go trials (SD = 71 ms), and −5 ms for stop trials (SD = 74 ms). Within stop-diastole trials, the mean timing for stop success trials was −4ms (SD = 76 ms), and for stop fail trials was −7ms (SD = 69 ms) (Supplementary Figure 1).

A total of 360 trials comprised 240 go trials (66%) and 120 stop trials (33%). On half of the stop trials, the stop cues were delivered at systole (290 ms after R-wave peak; ‘stop-systole’, n = 60), with the other half delivered at diastole (10 ms prior to R-wave peak; ‘stop-diastole’, n = 60). The stop signal delay (SSD) was adjusted on a trial-by-trial basis, using staircase tracking algorithms, to maintain stop success at 50%. Two separate staircase trackers were defined, one for stop-systole trials, and one for stop-diastole. Participants were instructed not to wait for stop cues and to respond as quickly and accurately to the direction of the arrow as possible. Stop signal reaction times (SSRTs), representing the internal response to the stop signal^20^, were calculated according to the integration method^21^.

We report statistical tests using repeated measures ANOVAs and t-tests to compare mean go RTs, SSRTs, and SSDs for systole and diastole trials, and to examine effect sizes according to η^2^ (ANOVA) and 95% confidence intervals of the mean difference (t-test), with JASP (version 0.7.5.5, JASP, 2016).

**Figure 2:**
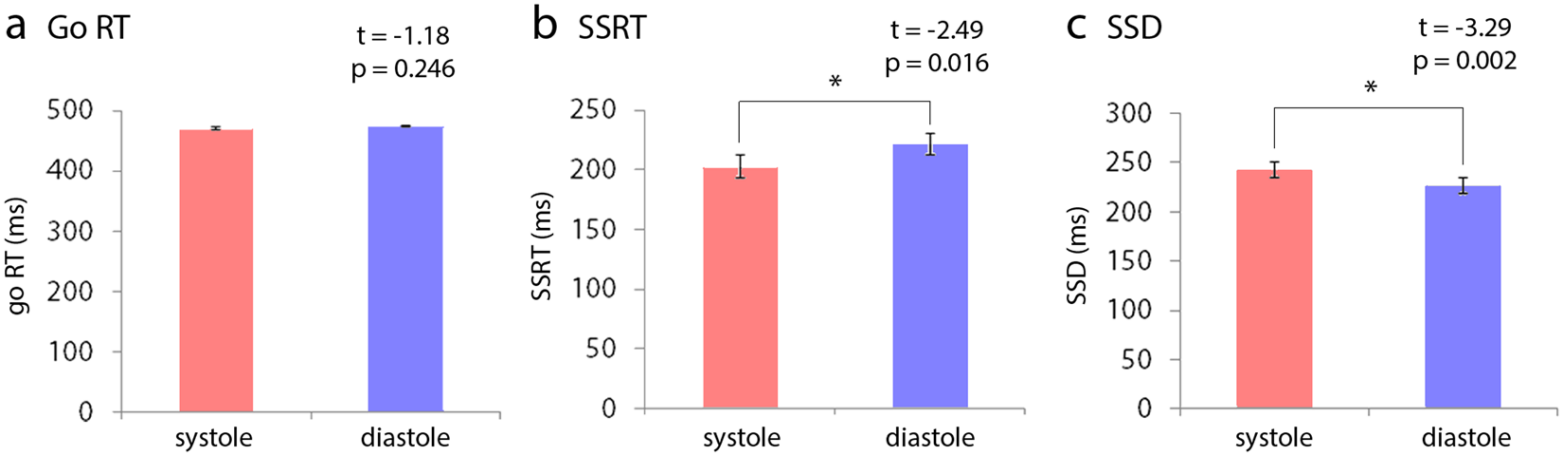
Cardiac cycle influences stopping ability. (**a**) Go RT is not impacted by cardiac timing, (**b**) SSRT is shorter at systole, (**c**) SSD is longer at systole. Error bars plotted using standard error of the mean. *Significant at p < 0.05.

Reaction times on go-systole trials (μ 471 ms) were not significantly different to go-diastole (μ 474 ms) (t(45) = −1.18, p = 0.246, μ difference = −3.30 [−8.96, 2.36]) (Fig. 2a). Response inhibition efficiency, however, as measured by the SSRT, was better at systole: SSRT-systole (203 ms) was shorter than SSRT-diastole (221 ms) (t(45) = −2.49, p = 0.016, μ difference = −18.37 [−33.22, −3.53]) (Fig. 2b). In addition, the SSD was longer at systole, indicating that during cardiac contraction participants tolerated longer stop signal delays at a 50% chance of successfully inhibiting their response: SSD-systole (243 ms) was longer than SSD-diastole (227 ms) (t(45) = −3.29, p = 0.002, μ difference = 16.40 [6.35, 26.44]) (Fig. 2c).

### Heart Rate Variability

To index baseline autonomic differences in sympathetic-parasympathetic balance, we calculated heart rate variability (HRV) during a quiet rest period of 2.5 minutes, with eyes open, during ECG recording. This also gave heart rate in beats per minute (mean 58bpm, SD = 10.05). A linear regression with HRV (as indexed by the root mean square of successive differences, RMSSD) as a dependent variable, and four response inhibition indices of SSRT-systole, SSRT-diastole, SSD-systole, and SSD-diastole as independent variables, suggested no relationship between baseline autonomic sympathetic-parasympathetic balance and overall task performance (F(4) = 1.28, p = 0.293; no significant coefficients).

**Figure 3:**
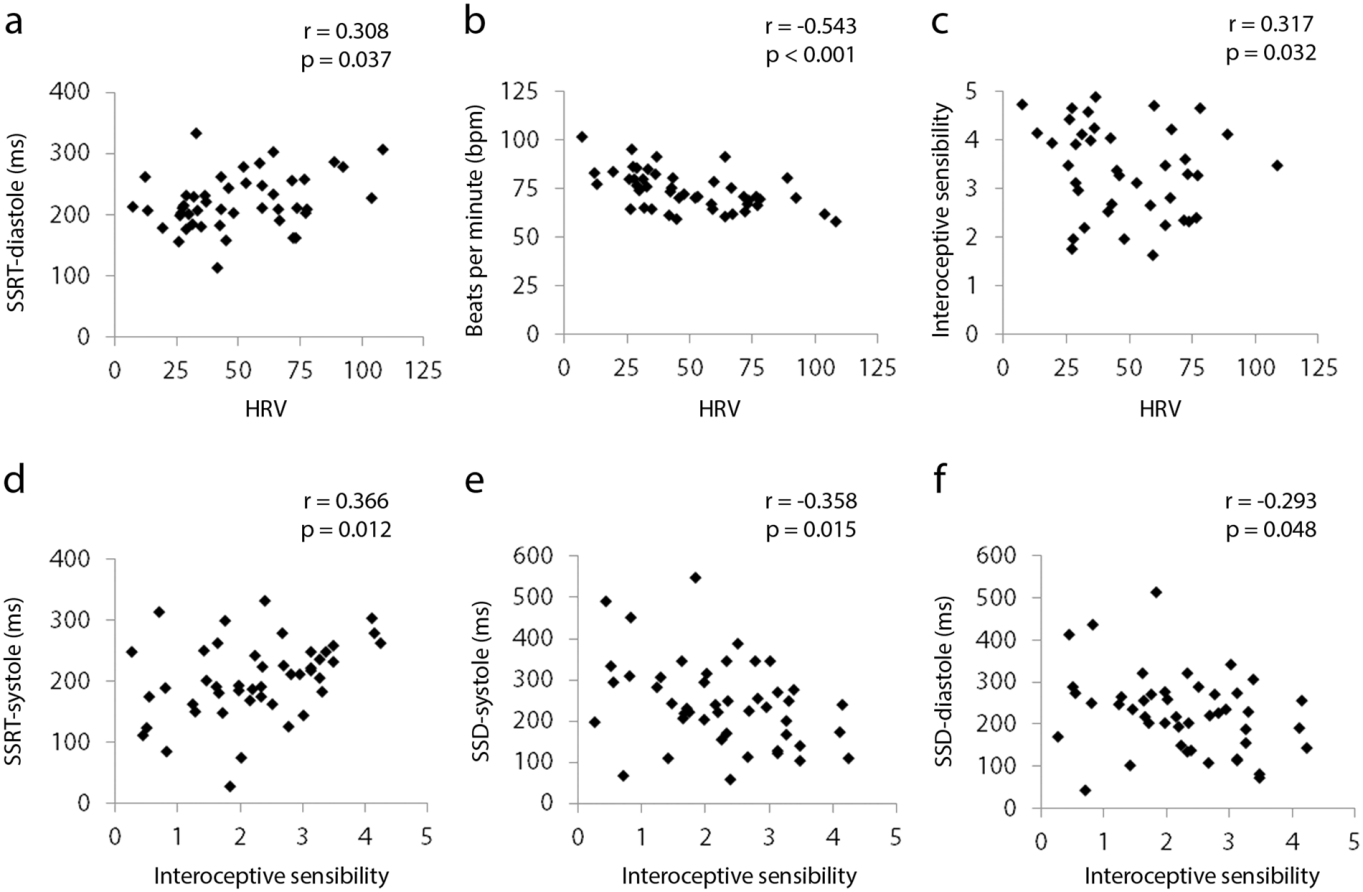
Individual differences in heart rate variability (HRV) and interoceptive sensibility. HRV correlates with (**a**) SSRT-diastole, (**b**) beats per minute (bpm), (**c**) interoceptive sensibility; interoceptive sensibility correlates with (**d**) SSRT-systole, (**e**) SSD-systole, (**f**) SSD-diastole (2-tailed tests). P values given prior to correction for multiple comparisons using false discovery rate (FDR) (see Table 1).

However, in an exploratory correlation analysis (Table 1) testing for 2-tailed correlations among HRV, beats per minute, and the four response inhibition indices, HRV correlated with (1) SSRT-diastole, such that the greater the HRV, the longer the SSRT (i.e. the poorer the response inhibition at diastole) (r = 0.308, p = 0.037); (2) beats per minute, such that the greater the HRV, the slower the heart rate (r = −0.543, p < 0.001), and (3) interoceptive sensibility, such that the greater the HRV, the greater the interoceptive sensibility (r = 0.317, p = 0.032) (Fig. 3a–c).

**Table 1:** Correlations (2-tailed) between cardiac stop signal task response inhibition indices and individual differences in cardiac physiology, trait impulsivity, and three dimensions of interoception.

	SSRT-s	SSRT-d	SSD-s	SSD-d	HRV	bpm	Barratt	Interoceptive accuracy	Interoceptive awareness	Interoceptive sensibility
SSRT-s										
SSRT-d	r −0.624 **p < 0**.**001** ***pFDR 0***.***006***									
SSD-s	r −0.830 **p < 0**.**001** ***pFDR 0***.***006***	r −0.633 **p < 0**.**001** ***pFDR 0***.***006***								
SSD-d	r −0.705 **p < 0**.**001** ***pFDR 0***.***006***	r −0.730 **p < 0**.**001** ***pFDR 0***.***006***	r 0.949 **p < 0**.**001** ***pFDR 0***.***006***							
HRV	r 0.144 p 0.450 pFDR 0.723	r 0.308 **p 0**.**037** pFDR 0.151	r −0.125 p 0.408 pFDR 0.706	r −0.164 p 0.276 pFDR 0.540						
bpm	r 0.118 p 0.435 pFDR 0.723	r −0.001 p 0.996 pFDR 0.996	r −0.032 p 0.832 pFDR 0.960	r −0.015 p 0.920 pFDR 0.966	r −0.543 **p < 0**.**001** ***pFDR 0***.***006***					
Barratt Impulsivity Scale	r 0.012 p 0.935 pFDR 0.966	r 0.073 p 0.630 pFDR 0.834	r −0.011 p 0.944 pFDR 0.966	r −0.052 p 0.731 pFDR 0.889	r 0.109 p 0.470 pFDR 0.729	r 0.211 p 0.158 pFDR 0.418				
Interoceptive accuracy	r −0.067 p 0.660 pFDR 0.849	r −0.201 p 0.180 pFDR 0.450	r 0.082 p 0.589 pFDR 0.828	r 0.156 p 0.302 pFDR 0.544	r −0.231 p 0.123 pFDR0.369	r −0.047 p 0.755 pFDR 0.894	r −0.281 p 0.059 pFDR 0.204			
Interoceptive awareness	r 0.018 p 0.908 pFDR 0.966	r −0.155 p 0.302 pFDR 0.544	r 0.076 p 0.618 pFDR 0.834	r 0.095 p 0.530 pFDR 0.795	r 0.016 p 0.917 pFDR 0.966	r 0.183 p 0.223 pFDR 0.478	r 0.220 p 0.143 pFDR 0.402	r 0.089 p 0.555 pFDR 0.806		
Interoceptive sensibility	r 0.366 **p 0**.**012** pFDR 0.068	r 0.175 p 0.246 pFDR 0.503	r −0.358 **p 0**.**015** pFDR 0.075	r −0.293 **p 0**.**048** pFDR 0.180	r 0.317 **p 0**.**032** pFDR 0.144	r −0.185 p 0.219 pFDR 0.478	r 0.063 p 0.679 pFDR 0.849	r −0.191 p 0.204 pFDR 0.478	r 0.258 p 0.084 pFDR 0.270	

Significant (p < 0.05) uncorrected correlations (p) indicated in bold, significant (p < 0.05) FDR corrected correlations (p_FDR_) indicated in *bold italics*.

Given we tested for several correlations between response inhibition indices and cardiac physiology, plus individual differences in interoception and impulsivity (see below), we corrected for multiple comparisons using false discovery rate (FDR) across all correlations, and report both FDR-adjusted and uncorrected p values (Table 1).

### Dimensions of Interoception

Individual participant interoceptive sensibility was calculated, along with interoceptive accuracy and interoceptive awareness, as three dimensions of interoception^22^. Interoceptive sensibility (subjectively perceived sensitivity to bodily sensations) was measured using the mean score of a participant’s responses to the 45 items of the Awareness section of the Body Perception Questionnaire^23^. Interoceptive accuracy was measured using a heartbeart tracking task^22,24^, in which participants were instructed to silently count the number of heartbeats perceived in a given interval, while a pulse oximeter measured objective heartbeats. Six trials with interval durations of 25, 30, 35, 40, 45 and 50 seconds were conducted in a randomised order. Following each trial, participants gave a confidence judgement in the perceived accuracy of their response, on a visual analogue scale (VAS) from ‘total guess/no heartbeat awareness’ to ‘complete confidence/full perception of heartbeat’. Interoceptive awareness was calculated according to the Pearson correlation between interoceptive accuracy and confidence rating^22^.

A series of linear regressions applied the three dimensions of interoception as dependent variables, and the four response inhibition indices as independent variables. Neither interoceptive sensibility (F(4) = 1.87, p = 0.135; no significant coefficients), interoceptive accuracy (F(4) = 0.94, p = 0.449; no significant coefficients), or interoceptive awareness (F(4) = 1.03, p = 0.406; no significant coefficients) related to task performance. However, in an exploratory correlation analysis (Table 1) testing for 2-tailed correlations among the three dimensions of interoception and response inhibition performance, interoceptive sensibility correlated with three of the four response inhibition indices: (1) SSRT-systole, such that the greater the interoceptive sensibility, the longer the SSRT at systole (r = 0.366, p = 0.012), (2) SSD-systole, such that the greater the interoceptive sensibility, the shorter the SSD at systole (r = −0.358, p = 0.015), and (3) SSD-diastole, such that the greater the interoceptive sensibility, the shorter the SSD at diastole (r = −0.293, p = 0.048), namely: the greater the interoceptive sensibility, the poorer the response inhibition, with greater evidence for this particularly at systole (Fig. 3d–f).

### Trait Impulsivity

To index impulsivity endophenotype, self-reported trait impulsivity was measured using the Barratt Impulsivity Scale^25^. However, a linear regression with trait impulsivity as a dependent variable, and the four response inhibition indices as independent variables, suggested no relationship between trait impulsivity and task performance (F(4) = 0.27, p = 0.898; no significant coefficients).

## Discussion

States of physiological arousal represent a potentially important influence on the capacity of an individual to inhibit behavioural responses. We tested whether response inhibition performance would change as a function of the cardiac cycle, during which transient physiological state cues might act as motivating guides for action. We observed that response inhibition efficiency was better, with shorter SSRT, at systole, when the heart is contracting (T-wave peak, approximately 300 ms following the R-wave), compared to when stop cues were presented at diastole, when the heart is relaxed between beats (R-wave peak). Furthermore, participants tolerated longer stop signal delays, at a 50% chance of successfully inhibiting their response, during systole. This suggests that response inhibition capacity is influenced by interoceptive physiological cues, such that transient signals from the heart (encoding cardiovascular arousal) aid motor control.

Furthermore, individual differences in heart physiology and perceived sensitivity to interoceptive cues were factors in the effect of cardiac timing on response inhibition. Participants with higher heart rate variability (HRV, which indexes dynamic sympathetic-parasympathetic balance) were more likely to show poorer response inhibition at diastole; while people with greater reported interoceptive sensibility were more likely to show poorer response inhibition particularly at systole. However, these relationships did not survive when applying stringent adjustment for multiple comparisons across the large number of exploratory correlation analyses. While further replicating samples are needed, these initial observations suggests that constitutional physiology and psychological aspects of interoceptive experience influence the impact of autonomic arousal cues on motor behaviour. These factors may form psychophysiological correlates of an impulsive endophenotype^19^.

At cardiac systole, baroreceptors in the aortic and carotid arteries signal ejection of blood from the heart, transmitting information about the timing and strength of heartbeats to cortical centres such as the insula, via pathways to brainstem, thalamus, and cortex^5–7^. Conversely, in between heartbeats, these arterial baroreceptors are quiescent. Heart rate is increased by sympathetic effects on the cardiac pacemaker and slowed by parasympathetic drive, hence systole (characterised by high baroreceptor firing) can arguably be viewed as a transient correlate of sympathetic arousal, while diastole (with low baroreceptor firing) parallels states of parasympathetic dominance. It is this latter state of cardiovascular ‘relaxation’ that we observe to be associated with poorer response inhibition.

Sub-second processing of physiological events, including cardiac contractions, permits brain centres to index threats and salient motivational challenges, in order to trigger rapid mitigating responses^2,3,5^. Here we find that momentary periods of reduced afferent physiological cues (which can be conceptualised as ‘somatic markers’^1^), when the heart is relaxed between beats, are associated with relatively poorer response inhibition.

Increasingly, a body of work suggests that sub-second perceptual and attentional processes may be altered across the cardiac cycle, modulating the saliency of relevant stimuli^3^. For example, at systole, there are lowered thresholds for breakthrough detection of subliminal fear stimuli^4^, and brief (supraliminal) fearful faces are perceived as more intense^4^. Historically, it was proposed that general sensory-motor processing would be enhanced during diastole^13^. However, this more recent work suggests a more complex interpretation: salient stimuli, such as facial signals of threat or fear, may be detected more readily at systole^4,26^, enabling prioritisation of adaptive behaviour, such as aversive action or even, as in the stop signal task, stopping of a motor act entirely.

Successful response inhibition on the stop signal task requires two primary and rapid processes: the detection of a salient cue, and the implementation of a stopping process. These processes may be mediated by interacting anatomical systems, encompassing insula, inferior frontal gyrus, pre-supplementary motor area, and basal ganglia nuclei, in particular the subthalamic nucleus^27–29^. Our task represents a novel examination of the interoceptive contribution to mechanisms of motor control. However, stop signal paradigms that control for attentional effects^30^ may be leveraged in combination with physiological state manipulations to dissect further mechanistic contributions to stopping processes.

Cognitive, neural, and physiological factors likely influence an individual’s tendency for impulsive behaviour^18,19^. We found that individuals with greater dynamic sympathetic-parasympathetic balance, as indexed by HRV, were more likely to show poorer response inhibition at diastole (prior to correction for multiple comparisons). This suggests that people with greater autonomic reactivity may be particularly poor at controlling impulsive motor responses during transient states of low arousal. Furthermore, a greater perceived sensitivity to bodily sensations, known as ‘interoceptive sensibility’^22^, was also associated with poorer response inhibition, particularly at systole (again prior to correction for multiple comparisons). In addition, HRV correlated with interoceptive sensibility: implying that these two factors have a common, or potentially interacting, effect on vulnerability to impulsive motor behaviour, although given the exploratory nature of the correlational analyses, we note this as an avenue for further exploration ahead of replicating samples. Furthermore, other measures of cardiovascular arousal, such as blood pressure, may be more informative indices for future studies with regards to impact that autonomic reactivity can have on impulsive behaviour.

Our insights have implications for treatment approaches to impulsivity behaviours. These include, for example, targeted modulation of monoaminergic transmission in pathways that selectively influence peripheral autonomic control via cardiac tonicity^31,32^, or biofeedback-based training enhancing interoceptive processes to increase sensitivity to transient changes in bodily arousal signals^33^. This interventional approach may be particularly relevant when individuals experience a mismatch between objective interoceptive cues, and perceived high sensitivity to such sensations^34^.

In our study, we focused on effects of interoceptive cues on motor behaviour. Since momentary cardiac deceleration is typically observed during response inhibition^15–17^, we separated each trial on the cardiac stop signal task with an inter-trial interval of 1000 ms, in addition to a period of 3 cardiac cycles over which the time of R-wave peak was dynamically monitored. This ensured delivery of task stimuli at desired points within the cardiac cycle, although we did not explicitly test for reliable, transitory effects that each task event had on cardiac cycle speed.

In addition, it is notable that while some previous studies have identified differences within the cardiac cycle on go reaction times^11,12^, we did not observe significant differences between systole and diastole on go trials. However, such previously reported effects have sometimes been small, and not present in all individuals^11^. It is also worth considering go reaction time analyses in the context of studies with go or choice reaction time tasks, versus motor inhibition tasks in which participants are aware they will be required to stop a motor response.

Although HRV and interoceptive sensibility may be predisposing factors to motor impulsivity, scores on the self-rated Barratt Impulsivity Scale (BIS) did not correlate with response inhibition performance (SSRTs and SSDs). Previous investigations do not always identify significant relationships between BIS scores and stopping ability^35^. Impulsivity subscale scores and mode of impulsivity may need to be considered to reveal relationships between trait impulsivity and task performance^36^.

Beyond cardiac cycle signals, other psychophysiological determinants shape the capacity to control action. Circadian rhythm preference (‘night owl’ versus ‘early bird’ typology) is associated with response prevention on the no-go task^37^, and medications that modulate bodily and cognitive arousal state improve performance on the stop signal task^38,39^. Future studies may usefully combine cardiac cycle task paradigms with pharmacological manipulations to characterise further the neurochemical mechanisms through which central and peripheral arousal interact to influence response inhibition capacity.

Signals from the heart and great arteries, typically signalling cardiovascular arousal, can act as motivating guides for action. Response inhibition capacity is reduced during periods of low afferent cardiovascular signalling, at cardiac diastole, and improved during transient putative states of physiological arousal, at cardiac systole. Aspects of heart physiology and perceived sensitivity to bodily sensations are putative factors in impulsive endophenotype vulnerability, influencing the mechanisms by which interoceptive cues support the expression of adaptive behaviour to salient events.

## Methods

### Participants

Sixty participants with no reported history of psychiatric or neurological disorders, and no intake of medications affecting neural or peripheral physiological function, were recruited from students and staff at the University of Sussex and Brighton and Sussex Medical School, and gave written informed consent to participate. The study was approved by the Brighton & Sussex Medical School Research Governance & Ethics Committee, and all research was performed in accordance with relevant regulations.

### Cardiac Stop Signal Task

The task was presented using Cogent2000 (version 1.32, http://www.vislab.ucl.ac.uk/cogent_2000.php) in Matlab (R2013a, Mathworks). Go cues, comprising black arrows pointing left and right, indicated a left or right button press to be made using the index and middle finger of the right hand respectively. On a minority (33%) of trials, after a variable stop signal delay (SSD), the black arrow was replaced by a red arrow and an auditory tone (1 kHz, 100 ms duration) was sounded, indicating participants should withhold their response on that trial. To maintain stop success at 50% the SSD was adjusted on a trial-by-trial basis, by subtraction of 50 ms after each incorrect response, and addition of 50 ms after each correct stop^21^. The starting SSD for the first stop trial was set at 200 ms for both stop-systole and stop-diastole staircase trackers. The go trials (black arrow go cue) ended as soon as participants responded, up to a maximum duration of 1000 ms. Similarly, stop trials (red arrow stop cue) ended as soon as participants responded, or on successful stop trials, ended at a maximum duration of 1000 ms.

To synchronise onset of the stop cues to specific time points within the cardiac cycle, we used ECG recording, with Cambridge Electronic Design (CED) hardware and Spike2 physiological recording software (version 7.17), interfacing cardiac events with the task in Matlab. An interactive threshold was applied to isolate each R-wave peak, with the inter-beat interval dynamically monitored in this way for 3 beats, giving a median inter-beat interval. This permitted the temporal prediction of the next R-wave peak and delivery of task cues at either cardiac systole, at 290 ms following R-wave peak, or cardiac diastole, at 10 ms prior to the R-wave peak. To establish the precision of trial event timing within the cardiac cycle, we used in-house Spike2 and Matlab scripts to extract the stimulus onsets from the Spike recording, and plot their relative position to the R-wave peak, in 50 ms time bins (Fig. 1d). Following the end of each trial, there was an inter-trial interval of 1000 ms, before the participant’s ECG was again monitored for 3 beats to establish the time of the next R-wave peak for the subsequent trial. This dynamic monitoring ensures accurate synchronisation of the task stimuli with desired cardiac events at all times throughout the duration of the task, given that a participant’s heart rate will vary over time, with attention, fatigue, and potentially, in response to stimuli and to actions^16^.

To present stop cues at systole or diastole, go cues on the stop trials were delivered at systole minus the SSD, or diastole minus the SSD: onset of the go cues within the cardiac cycle on stop trials was calculated dynamically for each trial, according to a prediction of the next R-wave, whether the trial was stop-systole or stop-diastole, using the current SSD according to the staircase trackers.

On 50% of go trials (n = 120), the onset of the go cues was at either systole (‘go-systole’, n = 60) or diastole (‘go-diastole’, n = 60). The remaining 50% of go trials (n = 120) were control trials, to control for the possibility that consistent timing of particular stimuli at certain points within the cardiac cycle can act as subliminal associative cues, indicating that subsequent events are likely to occur^40^: i.e. on stop trials, the onset of the go cue would arrive at one point in the cardiac cycle for stop-systole trials, and at another point for stop-diastole (bearing in mind a degree of variance according to changes in the SSD), while on go trials, go cues arrive at systole or diastole. These cues might serve to predict of what type the trial is, and whether a stop-systole or stop-diastole cue is potentially imminent. To mitigate this effect, we included control go trials, in which the onset of the go cue was set at the point within the cardiac cycle that go cues would be delivered on stop trials, using the values from the SSD staircase trackers, with no delivery of a subsequent stop cue. 50% of the control go trials (n = 60) used values from the SSD-systole tracker, and 50% (n = 60) from the SSD-diastole tracker. By using the SSD values which changed trial-to-trial according to the participant’s stopping performance, we varied the onset of the control go trials to further reduce the predictability of the go signals as subliminal cues for trial type.

The task was divided into four blocks of 90 trials. At the end of each block participants were permitted to take a rest break. This reduced movement during task trials and ensured high fidelity ECG recording uncontaminated by movement artefact. The time to complete the total 360 trials varied between participants according to their heart rate and how long they chose to rest between blocks, but was on average 35 minutes.

Stop signal reaction times (SSRTs) were calculated according to the integration method^21^, in which *n* go reaction times were rank ordered, and the SSD subtracted from the go reaction time corresponding to the n*probability of responding on stop trials. The SSD value from the SSD-systole and SSD-diastole trackers, and the probability of responding on stop-systole and stop-diastole trials, respectively, were used to calculate SSRT-systole and SSRT-diastole.

### Statistical Analysis

Regression analyses were run with default options in SPSS, using the ‘Enter’ method. In testing for several correlations between response inhibition indices, cardiac physiology, interoception, and impulsivity, we corrected p values (Table 1) for multiple comparisons using false discovery rate (FDR) across all correlations, in Matlab (Nantick 2013a) using a Matlab script by A. Winkler (https://s3.us-east-2.amazonaws.com/brainder/2011/fdr/fdr.m and described at https://brainder.org/2011/09/05/fdr-corrected-fdr-adjusted-p-values/)^41^.

### Data availability

The data analysed during this study are available from the corresponding author upon reasonable request.

### Code availability

The custom Spike2 and Matlab scripts to extract the stimulus onsets in ECG recording, and plot their relative position to the R-wave peak, are available from the corresponding author upon reasonable request.

## Electronic supplementary material

Supplementary Information
