# Supplementary material for: Response inhibition on the stop signal task improves during cardiac contraction
Source: Sci Rep. 2018 Jun 14;8:9136. doi: 10.1038/s41598-018-27513-y (PMC6002409; doi:10.1038/s41598-018-27513-y)
Supplement: Supplementary file 1 — Supplementary Information [file 41598_2018_27513_MOESM1_ESM.pdf]

Response inhibition on the stop signal task improves during cardiac contraction

SUPPLEMENTARY INFORMATION

Charlotte L Rae*[1,2], Vanessa E Botan[1,3], Cassandra D Gould van Praag[1,2], Aleksandra M Herman[3], Jasmina A K Nyyssönen[3], David R Watson[2], Theodora Duka[3], Sarah N Garfinkel[1,2,4], Hugo D Critchley[1,2,4]

[1] Sackler Centre for Consciousness Science, University of Sussex, UK
[2] Department of Neuroscience, Brighton & Sussex Medical School, UK
[3] School of Psychology, University of Sussex, UK
[4] Sussex Partnership NHS Foundation Trust, UK

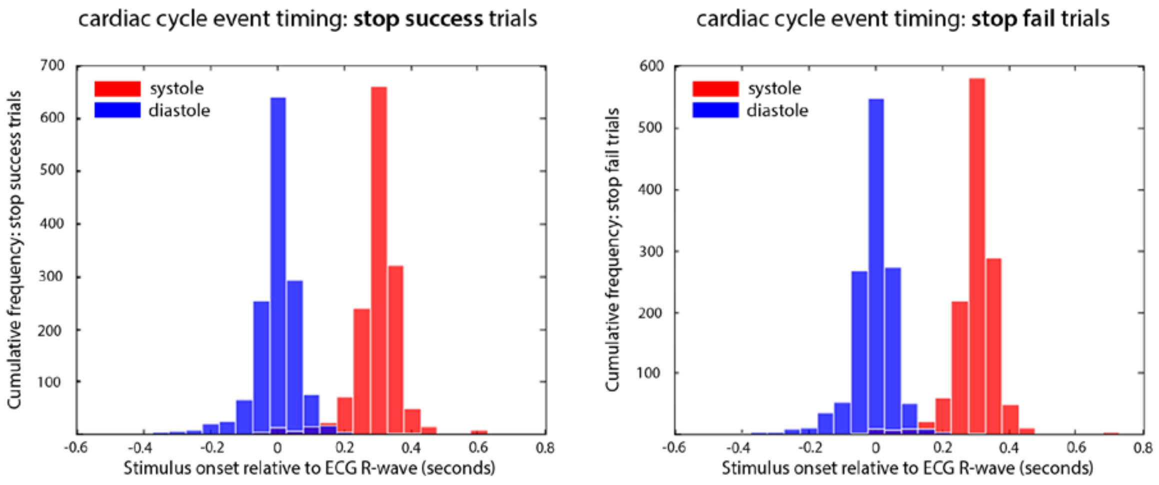

Supplementary Fig. 1 Cardiac cycle event timing on stop trials. Left: stop success (response withheld) trials; right: stop fail (button pressed) trials. Trial event timing within the cardiac cycle shown relative to the R-wave peak, in 50ms time bins: for both stop success and stop fail trials, >90% of trials were within 200ms of the intended timing for diastole trials at 10ms prior to the R-wave, and for systole trials at 290ms following R-wave. Dark blue indicates overlap of diastole and systole trial timings (minimal for trials of both stop outcomes).
